# Efficient regeneration of protoplasts from *Solanum lycopersicum* cultivar Micro-Tom

**DOI:** 10.1093/biomethods/bpae008

**Published:** 2024-02-06

**Authors:** Yeong Yeop Jeong, Yoo-Sun Noh, Suk Weon Kim, Pil Joon Seo

**Affiliations:** Department of Chemistry, Seoul National University, Seoul 08826, Korea; Plant Genomics and Breeding Institute, Seoul National University, Seoul 08826, Korea; Department of Biological Sciences, Sungkyunkwan University, Suwon 16419, Korea; School of Biological Sciences, Seoul National University, Seoul 08826, Korea; Biological Resource Center, Korea Research Institute of Bioscience and Biotechnology, Jeongeup 56212, Korea; Department of Chemistry, Seoul National University, Seoul 08826, Korea; Plant Genomics and Breeding Institute, Seoul National University, Seoul 08826, Korea; Department of Biological Sciences, Sungkyunkwan University, Suwon 16419, Korea

**Keywords:** Micro-Tom, protoplast regeneration, pluripotency, cell division, *de novo* organogenesis

## Abstract

Protoplast regeneration has become a key platform for genetic and genome engineering. However, we lack reliable and reproducible methods for efficient protoplast regeneration for tomato (*Solanum lycopersicum*) cultivars. Here, we optimized cell and tissue culture methods for protoplast isolation, microcallus proliferation, shoot regeneration, and plantlet establishment of the tomato cultivar Micro-Tom. A thin layer of alginate was applied to protoplasts isolated from third to fourth true leaves and cultured at an optimal density of 1 × 10^5^ protoplasts/ml. We determined the optimal culture media for protoplast proliferation, callus formation, *de novo* shoot regeneration, and root regeneration. Regenerated plantlets exhibited morphologically normal growth and sexual reproduction. The entire regeneration process, from protoplasts to flowering plants, was accomplished within 5 months. The optimized protoplast regeneration platform enables biotechnological applications, such as genome engineering, as well as basic research on plant regeneration in *Solanaceae* species.

## Introduction

Plants have evolved the remarkable ability to reprogram their cells and can regenerate tissues, organs, or plantlets, even from single cells [1, 2]. This regeneration capacity has been widely used for *in vitro* protoplast regeneration. Protoplast regeneration involves a multi-step process of cell wall recovery, cell cycle reentry, pluripotent callus proliferation, shoot regeneration, *de novo* root regeneration, and plantlet establishment [3–7]. As protoplasts are important plant materials for genome engineering [8–10], the development of efficient and reliable methodologies for protoplast regeneration is essential. The clustered regularly interspaced short palindromic repeat (CRISPR)/CRISPR-associated protein 9 (Cas9) system has been transiently expressed in protoplasts from diverse plant species via DNA-free methods [11], and genome-edited protoplasts have been regenerated into individual plants [11–17]. However, protoplast regeneration remains challenging for many important cultivars of crop and model species.

Tomato (*Solanum lycopersicum*) is an important vegetable crop [12] and one of the best-studied cultivated dicotyledonous plants [18]. The tomato cultivar Micro-Tom has been widely used because of its small genome size (2*n *=* *24), dwarf and compact growth phenotypes, and short life cycle, and it is recognized as a research model for *Solanum* species [19, 20]. Genome engineering techniques have been extensively applied to protoplasts of various *Solanum* species [21–28]. However, despite high demand for genome engineering in *Solanum* species, a reliable method for protoplast regeneration still needs to be established for basic research as well as biotechnological applications in *Solanum* relatives.

Protoplast regeneration procedures vary by species [29]. While regeneration methods have been developed for several plant species, including *Solanum* species [30–36], a complete protoplast regeneration method for tomato cultivar Micro-Tom remains to be developed. Here, based on recent advances in alginate gel methods using calcium ions (Ca^2+^) and alginate [3], we optimized the Micro-Tom protoplast regeneration protocol by optimizing the tissue types for protoplast isolation as well as cell culture media and conditions. Moreover, the entire protoplast regeneration process was completed within 5 months. Plantlets derived from protoplasts were fertile and produced morphologically normal progeny. This optimized protoplast regeneration technique will facilitate fundamental investigations of pluripotency in tomato and biotechnical applications such as genome engineering.

## Materials and methods

### Chemicals and equipment

All chemicals were purchased from Sigma-Aldrich, Duchefa Biochemie, Merck, Novozymes, or Junsei (Supplementary Table S1). The equipment used in this study is listed in Supplementary Table S2.

### Solutions and culture media

- MMC solution: 0.47 M Mannitol, 10 mM 2-morpholinoethanesulfonic acid monohydrate (MES·H_2_O), 10 mM CaCl_2_ were prepared in double distilled water (ddH_2_O). The pH was adjusted to 5.8 with 2 M NaOH and/or 1 M HCl. The solution was sterilized in an autoclave at 121°C for 10 min and stored at room temperature.
- Enzyme solution: 2% (v/v) Viscozyme L, 1% (v/v) Celluclast 1.5 L, and 1% (v/v) Pectinex ultra SP-L were prepared in MMC solution. The solution was sterilized using a 0.2-µm syringe filter.
- 0.6 M Surcose solution: 0.6 M Sucrose and 2 mM MES·H_2_O were prepared in ddH_2_O. The pH was adjusted to 5.8 with 2 M NaOH and/or 1 M HCl. The solution was sterilized in an autoclave at 121°C for 10 min and stored at room temperature.
- 0.5 M Mannitol solution: 0.5 M Mannitol and 2 mM MES·H_2_O were prepared in ddH_2_O. The pH was adjusted to 5.8 with 2 M NaOH and/or 1 M HCl. The solution was sterilized in an autoclave at 121°C for 10 min and stored at room temperature.
- 2.8% (w/v) sodium alginate solution: 2.8% (w/v) sodium alginate and 0.4 M Mannitol were prepared in ddH_2_O. The solution was sterilized in an autoclave at 121°C for 10 min and stored at room temperature. Any precipitates in the sodium alginate solution were removed using a 0.2-µm syringe filter.
- CaCl_2_-agar: 20 mM CaCl_2_ and 0.4 M Mannitol were prepared in ddH_2_O. The 1% (v/v) plant agar was added to the prepared solution. The final solution was autoclaved at 121°C for 10 min and stored at 4°C.
- CaCl_2_ solution: 50 mM CaCl_2_ and 0.4 M Mannitol were prepared in ddH_2_O. The solution was sterilized in an autoclave at 121°C for 10 min and stored at room temperature.
- Cell and tissue culture media: All liquid media were sterilized using a 0.2-µm syringe filter. The composition of all solutions and culture media is summarized in Table 1.

**Table 1.** Composition of culture media for *S. lycopersicum* cv. Micro-Tom protoplast regeneration.

Medium name	Medium composition	Storage	Function	References
Protoplast induction medium–high cytokinin concentration (*Sl*-PIM-hCk)	Gamborg B5 medium containing vitamins, 30 g/l sucrose, 60 g/l *myo*-inositol, 2 mg/l 6-BAP, and 0.5 mg/l α-NAA (pH adjusted to 5.8 using 2 M NaOH or 1 M HCl). Medium sterilized using a 0.2-µm syringe filter	Freshly prepared	Induction of protoplast division	[3]
Protoplast induction medium–high auxin concentration (*Sl*-PIM-hAx)	Gamborg B5 medium containing vitamins, 30 g/l sucrose, 60 g/l *myo*-inositol, 0.5 mg/l *trans*-zeatin, and 2.0 mg/l α-NAA (pH adjusted to 5.8 using 2 M NaOH or 1 M HCl). Medium sterilized using a 0.2-µm syringe filter	Freshly prepared	Induction of protoplast division	This study
LCM	Gamborg B5 medium without NH_4_NO_3_, KM-8P vitamins, 0.88 mg/l folic acid, 70 g/l d-mannitol, 10 g/l sucrose, 5 g/l d-glucose, 0.1 g/l casein hydrolysate, 1 mg/l α-NAA, 0.5 mg/l 2,4-D, and 0.5 mg/l 6-BAP (pH adjusted to 5.8 using 2 M NaOH or 1 M HCl). Medium sterilized using a 0.2-µm syringe filter	Freshly prepared	Induction of protoplast division	[32]
Callus induction medium–high cytokinin concentration (*Sl*-CIM-hCk)	Gamborg B5 medium containing vitamins, 30 g/l sucrose, 2 mg/l 6-BAP, and 0.5 mg/l α-NAA (pH adjusted to 5.8 using 2 M NaOH or 1 M HCl). Medium sterilized using a 0.2-µm syringe filter	Freshly prepared	Microcallus growth	[3]
Callus induction medium–high auxin concentration (*Sl*-CIM-hAx)	Gamborg B5 medium containing vitamins, 30 g/l sucrose, 0.5 mg/l *trans*-zeatin, and 2.0 mg/l α-NAA (pH adjusted to 5.8 using 2 M NaOH or 1 M HCl). Medium sterilized using a 0.2-µm syringe filter	Freshly prepared	Microcallus growth	This study
LCM-nAx	Gamborg B5 medium without NH_4_NO_3_, KM-8P vitamins, 0.88 mg/l folic acid, 70 g/l d-mannitol, 10 g/l sucrose, 5 g/l d-glucose, 0.1 g/l casein hydrolysate, and 0.75 mg/l 6-BAP (pH adjusted to 5.8 using 2 M NaOH or 1 M HCl). Medium sterilized using a 0.2-µm syringe filter	Freshly prepared	Microcallus growth	[32]
Shoot induction medium (*Sl*-SIM-J)	MS medium containing vitamins, 30 g/l sucrose, 0.47 g/l MES·H_2_O, 0.1576 mg/l IAA, and 0.501 mg/l 2-IP. pH adjusted to 5.8 using 2 M NaOH or 1 M HCl before the addition of plant agar (8 g/l). Medium sterilized by autoclaving at 121°C for 10 min	Freshly prepared	Induction of shoot regeneration	[3]
Shoot induction medium (*Sl*-SIM-T)	MS medium containing vitamins, 30 g/l sucrose, 0.47 g/l MES·H_2_O, 2 mg/l *trans*-zeatin, and 0.1 mg/l IAA. pH adjusted to 5.8 using 2 M NaOH or 1 M HCl before the addition of plant agar (8 g/l). Medium sterilized by autoclaving at 121°C for 10 min	Freshly prepared	Induction of shoot regeneration	[32]
Shoot induction medium (*Sl*-SIM-N)	MS medium containing vitamins, 10 g/l sucrose, 0.47 g/l MES·H_2_O, 2 mg/l *trans*-zeatin, 0.01 mg/l α-NAA, and 0.1 mg/l GA3. pH adjusted to 5.8 using 2 M NaOH or 1 M HCl before the addition of plant agar (8 g/l). Medium sterilized by autoclaving at 121°C for 10 min	Freshly prepared	Induction of shoot regeneration	[44]
Shoot induction medium (*Sl*-SIM-A)	MS medium containing vitamins, 20 g/l d-glucose, 0.47 g/l MES·H_2_O, 0.75 mg/l *trans*-zeatin, and 0.1 mg/l ΙΑΑ. pH adjusted to 5.8 using 2 M NaOH or 1 M HCl before the addition of plant agar (8 g/l). Medium sterilized by autoclaving at 121°C for 10 min	Freshly prepared	Induction of shoot regeneration	[17]
Shoot induction medium (*Sl*-SIM-V)	Gamborg B5 medium containing vitamins, 20 g/l d-glucose, 0.47 g/l MES·H_2_O, 0.15 mg/l IAA, and 5 mg/l 2-IP. pH adjusted to 5.8 using 2 M NaOH or 1 M HCl before the addition of plant agar (8 g/l). Medium sterilized by autoclaving at 121°C for 10 min	Freshly prepared	Induction of shoot regeneration	[45]
Murashige and Skoog medium (*Sl*-MS)	1/2 MS medium containing vitamins, 30 g/l sucrose, and 0.47 g/l MES·H_2_O. pH adjusted to 5.8 using 2 M NaOH or 1 M HCl before the addition of plant agar (8 g/l). Medium sterilized by autoclaving at 121°C for 10 min	Freshly prepared	Induction of root emergence; seedling growth	[3]
Rooting medium (*Sl*-RM)	1/2 MS medium containing vitamins, 30 g/l sucrose, 0.47 g/l MES·H_2_O, and 1 mg/l IBA. pH adjusted to 5.8 using 2 M NaOH or 1 M HCl before the addition of plant agar (8 g/l). Medium sterilized by autoclaving at 121°C for 10 min	Freshly prepared	Induction of root emergence	[3]

### Plant materials and growth conditions

Tomato (*S. lycopersicum*) cultivar Micro-Tom was used.

1. Soak Micro-Tom seeds in 1 ml 70% (v/v) ethanol for 2 min. Replace the ethanol with 1 ml 25% (v/v) commercial bleach containing 0.1% (v/v) Tween-20 for 20 min. Rinse the seeds with 1 ml of sterile distilled water five times. After drying, sow the sterilized seeds on *Sl*-MS medium (100-mm culture plate) and incubate at 25°C under dark conditions for 3 days.
2. Grow seedlings at 25°C under a long-day (LD) photoperiod (16 h light/8 h dark) with 70 µmol photons/m^2^/s light intensity provided by cool-white fluorescent lamps.

### Cell wall digestion of true leaves from Micro-Tom

**Figure 1:**
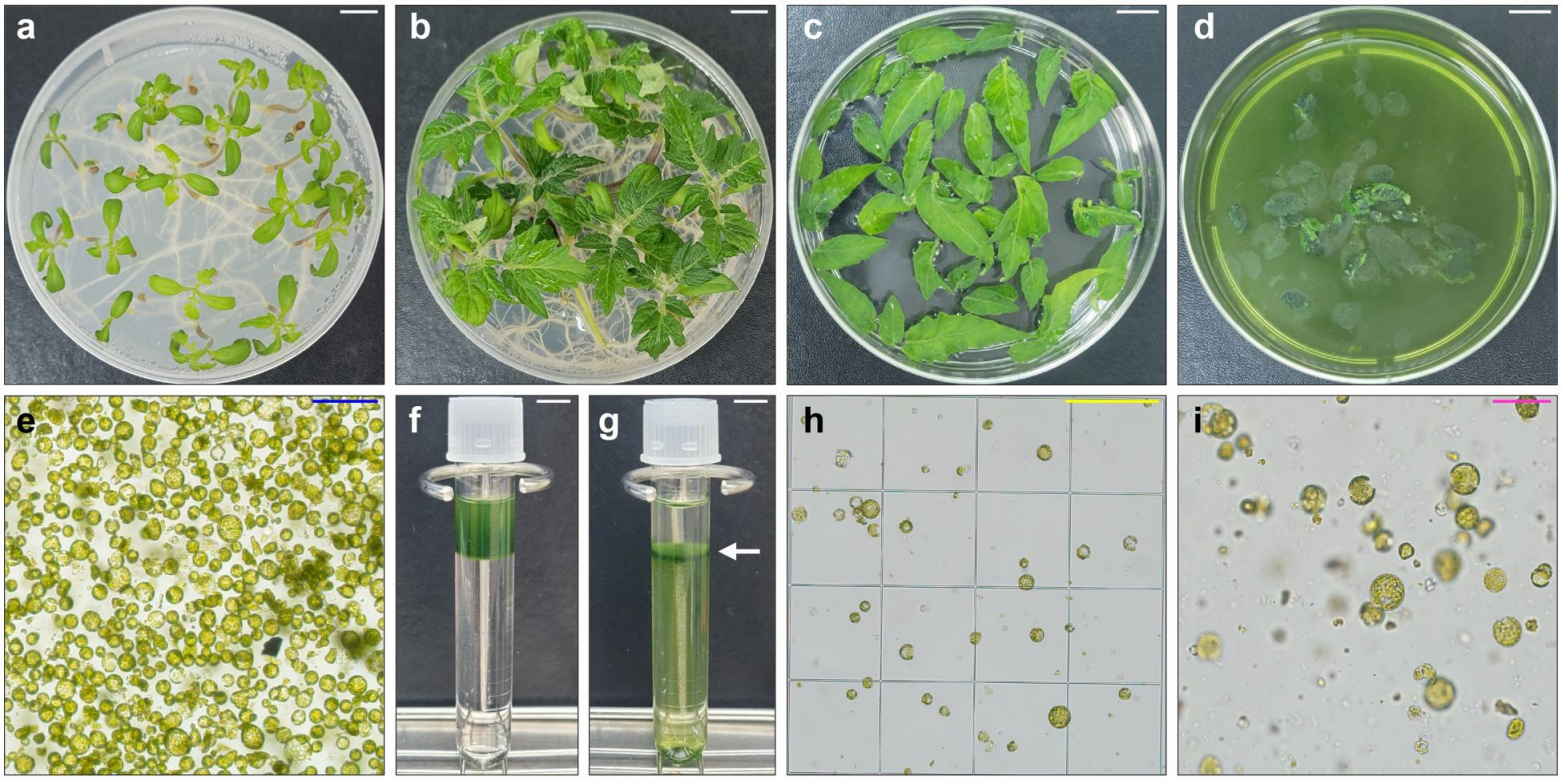
Protoplast isolation from true leaves of *S. lycopersicum* cv. Micro-Tom. (**a** and **b**) Ten-day-old (**a**) and 20-day-old (**b**) seedlings grown under LD (16 h light/8 h dark) conditions. (**c**) Pre-plasmolysis of true leaves in 0.5 M mannitol. (**d**) Protoplast isolation at 6 h post-incubation in enzyme solution. (**e**) Microscope image of isolated protoplasts at 6 h post-incubation in enzyme solution. (**f**) Protoplast solution overlaid onto 0.6 M sucrose during sucrose density gradient-based protoplast purification. (**g**) Viable protoplasts in the middle of the sucrose gradient solution after centrifugation. Arrow indicates the protoplast layer. (**h**) Microscope image of purified protoplasts using a hemocytometer. (**i**) Protoplasts in PIM-incubated alginate hydrogel. White bars = 1 cm; yellow bar = 1 mm; blue bar = 100 µm; magenta bar = 50 µm

Protoplast isolation is an important first step in the protoplast regeneration protocol and determines protoplast yield, quality, and regeneration capacity. We prepared protoplasts from two different plant tissues, cotyledons from 10-day-old seedlings and true leaves from 20-day-old seedlings (Fig. 1a and b), to determine the optimal source for protoplast isolation. Plasmolysis treatment of plant tissues was conducted in 0.5 M mannitol for 15–20 min to ensure high protoplast yield (Fig. 1c). Pre-plasmolysed explants were incubated in enzyme solution for 5–6 h with gentle shaking (Fig. 1d and e).

We confirmed the yield of intact protoplasts in the range of 2–3 × 10^7^ protoplasts/g fresh weight (FW) of true leaves or 3–4 × 10^6^ protoplasts/g FW of cotyledons after incubation in 20 ml of enzyme solution for 6 h. Considering that the isolation of a large number of viable protoplasts is important for successful protoplast regeneration, true leaves were chosen as the source for Micro-Tom protoplast isolation and subsequent culture.

#### Protoplast isolation protocol

All of the following steps should be conducted under sterile conditions, and all solutions and materials must be sterilized to avoid contamination.

1. For pre-plasmolysis treatment, use forceps to transfer 30–40 true leaves from 3-week-old seedlings to a 90-mm Petri dish containing 20 ml 0.5 M mannitol; seal the Petri dish with Parafilm.
2. Incubate the Petri dish in the dark at 25°C for 15–20 min without shaking.
3. Replace the 0.5 M mannitol with 20 ml enzyme solution and seal the Petri dish with Parafilm.
4. Incubate the Petri dish at 25°C in darkness for 5–6 h, with gentle shaking at 60 rpm.
*Note 1:* Extended incubation in enzyme solution leads to fragile protoplasts. When incubation time exceeds 8 h, breakage and dysfunction of protoplasts occurs. Therefore, incubation for 5–6 h is recommended.
*Note 2:* The yield of intact protoplasts is in the range of 2–3 × 10^7^ protoplasts/g FW of true leaves.

### Protoplast culture in Ca^2+^-alginate hydrogels

Only a small portion of isolated Micro-Tom protoplasts re-entered the cell cycle. To enrich the culture for viable protoplasts with cell division potential, we conducted sucrose density gradient purification (Fig. 1f and g). Since we recently found that protoplasts embedded in hydrogels, especially Ca^2+^-alginate hydrogel, usually display greater cell survival and proliferation [3, 4, 37, 38], we employed a protoplast-embedding method using a Ca^2+^-alginate hydrogel and placed 2 ml of protoplast–alginate mixture onto a 60-mm CaCl_2_-agar plate, resulting in hydrogels with a uniform diameter (60 mm) and thickness (0.5 mm) (Fig. 1h and i).

#### Protoplast purification and embedding protocol

1. Filter protoplasts immersed in 20 ml enzyme solution using 40-µm cell strainers to remove undigested tissues and debris; collect the filtrate in a 90-mm Petri dish.
*Note:* Examine the filtered protoplasts under a light microscope to confirm yield and intactness.
2. Split the filtrate equally between two 14-ml round-bottom tubes; adjust the final volume in each tube to 12 ml using MMC solution.
3. Centrifuge the tubes using a swinging-bucket rotor at 80*g* for 7 min at room temperature.
4. Remove the supernatant.
*Note:* The supernatant does not need to be completely removed. A residual volume of <100 µl is acceptable.
5. Carefully resuspend the pellet in each tube using 1 ml MMC solution.
6. Add 6 ml 0.6 M sucrose to each of two new 14-ml round-bottom tubes.
7. For each sample, carefully overlay 2 ml protoplast suspension onto 0.6 M sucrose in one of the new tubes.
8. Centrifuge the samples using a swinging-bucket rotor at 80*g* for 10 min at room temperature.
9. For each sample, transfer 2 ml purified protoplasts into a new 14-ml round-bottom tube; intact protoplasts will be suspended at the sucrose–MMC interface.
10. Adjust the volume in each tube to 5 ml using 0.5 M mannitol, and resuspend the protoplasts.
11. Centrifuge the samples using a swinging-bucket rotor at 80*g* for 5 min at room temperature.
12. Remove the supernatant.
13. Resuspend the protoplasts in each tube in 5 ml 0.5 M mannitol; estimate the number of protoplasts in each tube under a microscope using a hemocytometer.
14. Centrifuge the samples using a swinging-bucket rotor at 80*g* for 5 min at room temperature.
15. Remove the supernatant.
*Note:* The supernatant should be removed completely. Residual Ca^2+^ ions present in the MMC solution will result in premature polymerization of the protoplast–alginate mixture.
16. Resuspend the protoplasts in 0.5 M mannitol to 2 × 10^5^ protoplasts/ml.
17. Mix 1 ml protoplast suspension (2 × 10^5^ protoplasts) gently with 1 ml 2.8% (w/v) sodium alginate solution for alginate hydrogel formation.
*Note:* The final protoplast density in the protoplast–alginate mixture should be in the range of 0.5–1 × 10^5^ protoplasts/ml.
18. Pour the 2 ml protoplast–alginate mixture onto CaCl_2_-agar in a 60-mm Petri dish; incubate the plate at room temperature for 1 h.
*Note:* The alginate layer should be uniformly thin (∼0.5 mm) across the agar plate.
19. Pour 2 ml CaCl_2_ solution onto the alginate hydrogel; incubate the plate for 30 min at room temperature to completely solidify the gel.

### Protoplast division and microcallus formation

**Figure 2:**
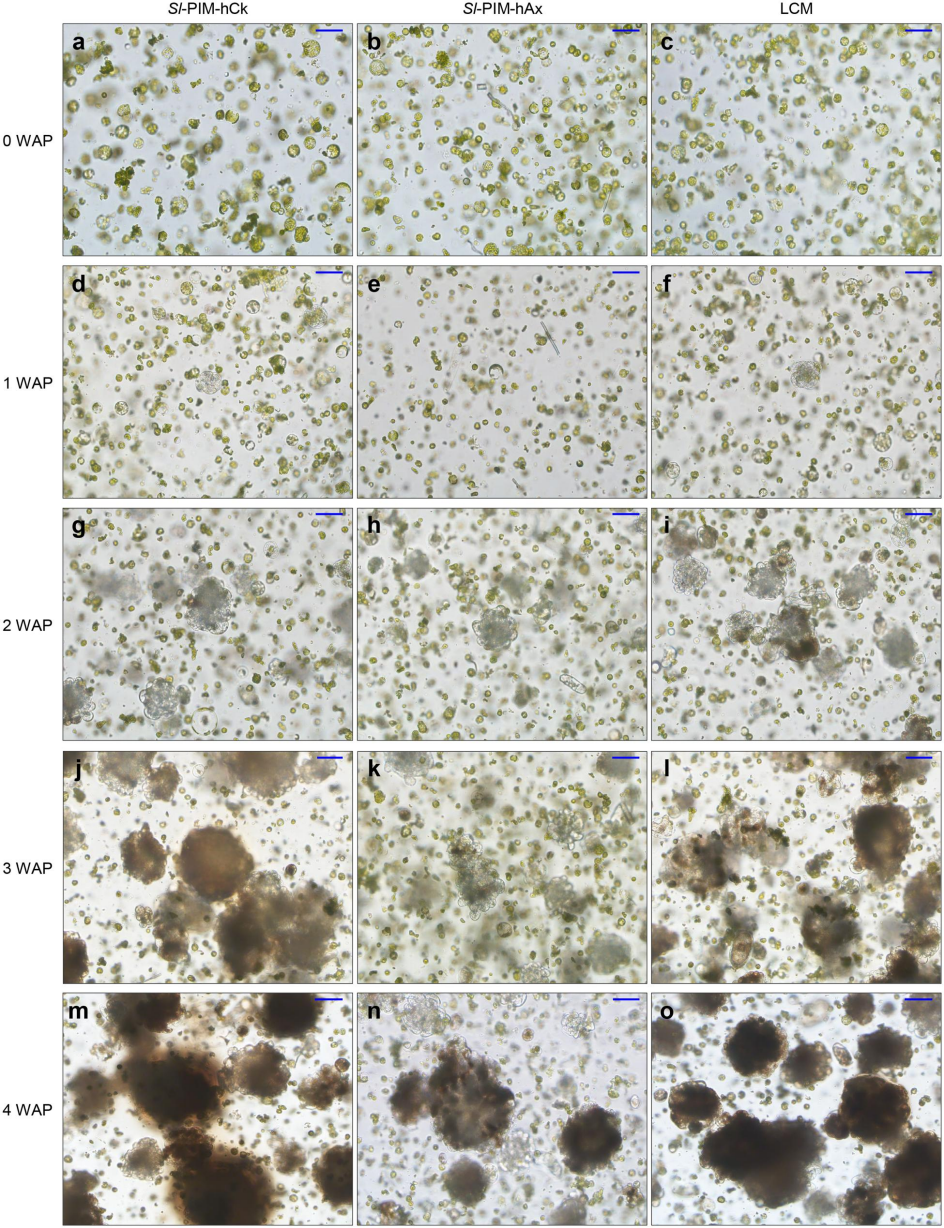
Division of protoplasts derived from true leaves in three different protoplast proliferation media. (**a**–**o**) Images of protoplasts taken at the indicated number of WAP: 0 WAP (**a**–**c**), 1 WAP (**d**–**f**), 2 WAP (**g**–**i**), 3 WAP (**j**–**l**), and 4 WAP (**m**–**o**). Bars = 100 µm

The re-entry of protoplasts into the cell cycle is important for microcallus formation [39]. We observed the first cell division of protoplasts at ∼1 week after incubation in a protoplast proliferation medium (WAP) (Fig. 2a–f), although only a small fraction of protoplasts divided. Proliferating protoplasts (diameter ≈100 µm) were observed at ∼2 WAP (Fig. 2g–i). The protoplasts grew into microcalli (Fig. 2j–l), which reached 0.3 mm in diameter at ∼4 WAP (Fig. 2m–o), while most protoplasts remained in a nonproliferative state.

To determine the optimal culture medium for proliferation of Micro-Tom protoplasts, we measured protoplast proliferation rates in three different liquid media: *Sl*-PIM-hCk, *Sl*-PIM-hAx, and lycopersicon culture medium (LCM) [3, 32] (Fig. 2 and Table 2; Supplementary Fig. 1). Protoplasts cultured in *Sl*-PIM-hCk or LCM exhibited a high proliferation rate, and those cultured in *Sl*-PIM-hAx showed a relatively low proliferation rate. At the later stages of protoplast division, proliferation efficiency was higher in *Sl*-PIM-hCk than in *Sl*-PIM-hAx (Fig. 2m and n).

**Table 2.** Division efficiency of protoplasts of *S. lycopersicum* cv. Micro-Tom in three different media.

Culture date	*Sl*-PIM-hCk	*Sl*-PIM-hAx	LCM
	(protoplasts/mm^2^)	(protoplasts/mm^2^)	(protoplasts/mm^2^)
2 WAP	12.88 ± 1.56	6.41 ± 0.96	11.65 ± 1.63
3 WAP	22.53 ± 1.59	14.4 ± 1.69	19.23 ± 1.50

Protoplasts were embedded in Ca^2+^-alginate hydrogels, which were incubated in three different media for 2 and 3 weeks. Each alginate hydrogel contains a concentration of 1 × 10^5^ protoplasts/ml. The average number of proliferating protoplasts was calculated by counting dividing protoplasts in a unit area (1.99 mm^2^) of hydrogel. Data represent the mean ± SEM of biological triplicates.

While protoplast proliferation media were suitable for cell division at the initial stages of protoplast proliferation, they were sub-optimal for microcallus formation. A gradual reduction of the osmotic pressure in protoplast division medium is necessary for promoting continuous cell proliferation [40, 41]. Thus, after 4 weeks of incubation, the protoplast proliferation medium should be replaced with the medium of a similar composition but of a lower osmotic pressure.

**Figure 3:**
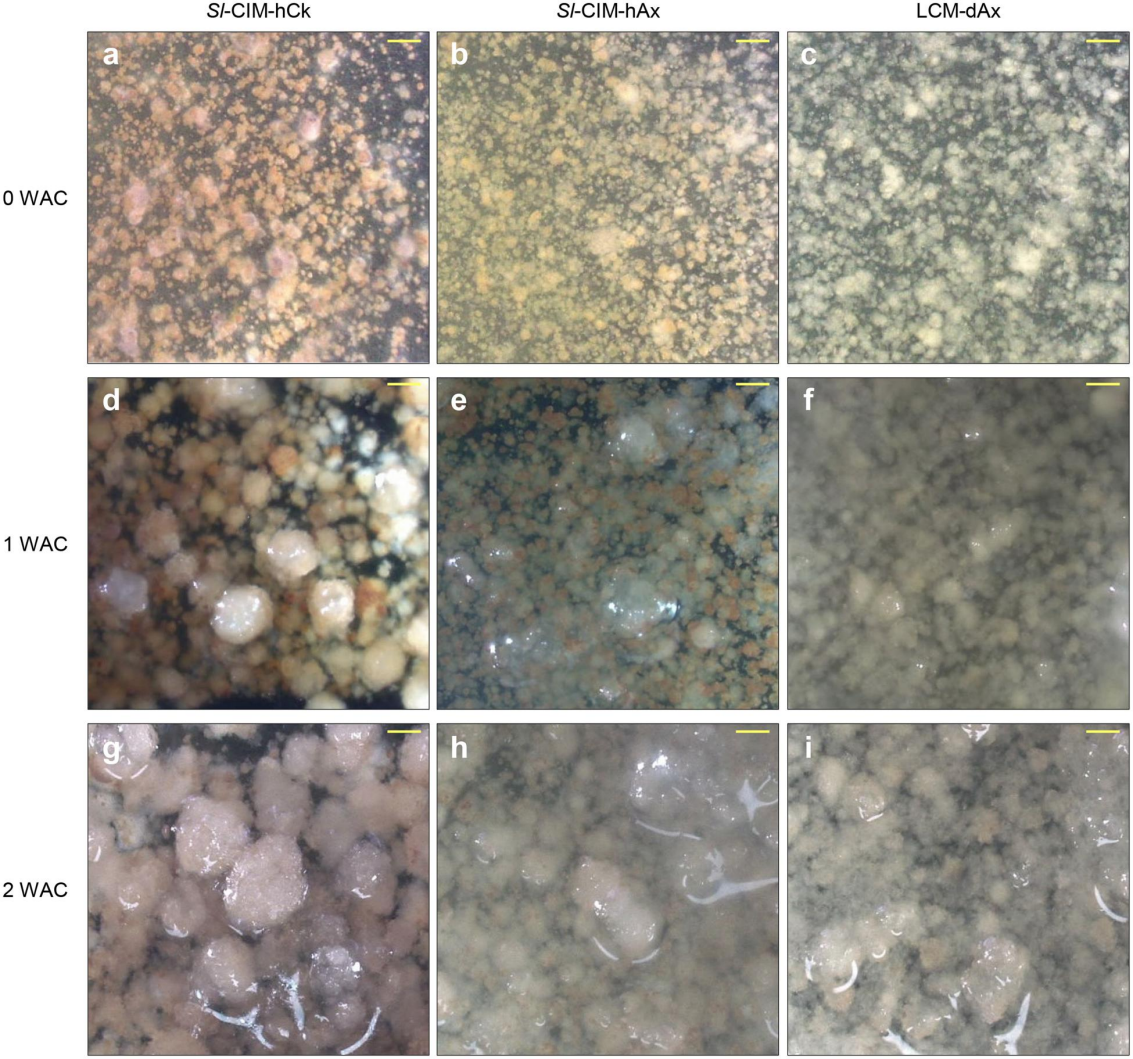
Microcallus formation in three different callus proliferation media. (**a**–**i**) Images of callus taken at the indicated number of WAC: 0 WAC (**a**–**c**), 1 WAC (**d**–**f**), and 2 WAC (**g**–**i**). Bars = 1 mm

We examined three types of callus proliferation media: *Sl*-CIM-hCk (for protoplasts incubated in *Sl*-PIM-hCk during protoplast proliferation), *Sl*-CIM-hAx (for protoplasts incubated in *Sl*-PIM-hAx), and LCM-nAx (for protoplasts incubated in LCM) (Fig. 3) [3, 32]. After 2 weeks of incubation, protoplasts cultured in *Sl*-CIM-hCk showed a higher callus formation efficiency than those cultured in *Sl*-CIM-hAx or LCM-nAx. The combination of *Sl*-PIM-hCk and *Sl*-CIM-hCk was also good for the subsequent shoot regeneration (Supplementary Fig. S2). We therefore recommend *Sl*-PIM-hCk and *Sl*-CIM-hCk for the highest callus formation efficiency.

#### Microcallus formation protocol

1. Cut the protoplast–alginate gel into four pieces using a sterilized scalpel blade; transfer one piece of gel to a 60-mm Petri dish containing 4 ml liquid *Sl*-PIM-hCk using a sterilized spatula.
2. Seal the plates with Parafilm and incubate the protoplast–alginate gel at 25°C in darkness for 4 weeks. Exchange the liquid *Sl*-PIM-hCk with 4 ml fresh liquid *Sl*-PIM-hCk every week.
*Note:* After 2 weeks of incubation in *Sl*-PIM-hCk, proliferating protoplast colonies reach 100∼200 μm in diameter.
3. Replace *Sl*-PIM-hCk with 4 ml *Sl*-CIM-hCk.
4. Incubate the alginate gel at 25°C in darkness for 2 weeks. Exchange the *Sl*-CIM-hCk with 4 ml fresh *Sl*-CIM-hCk every week.
*Note:* Most microcalli should be at least 0.8 mm in diameter before inducing *de novo* shoot formation.

### Shoot regeneration from microcalli

**Figure 4:**
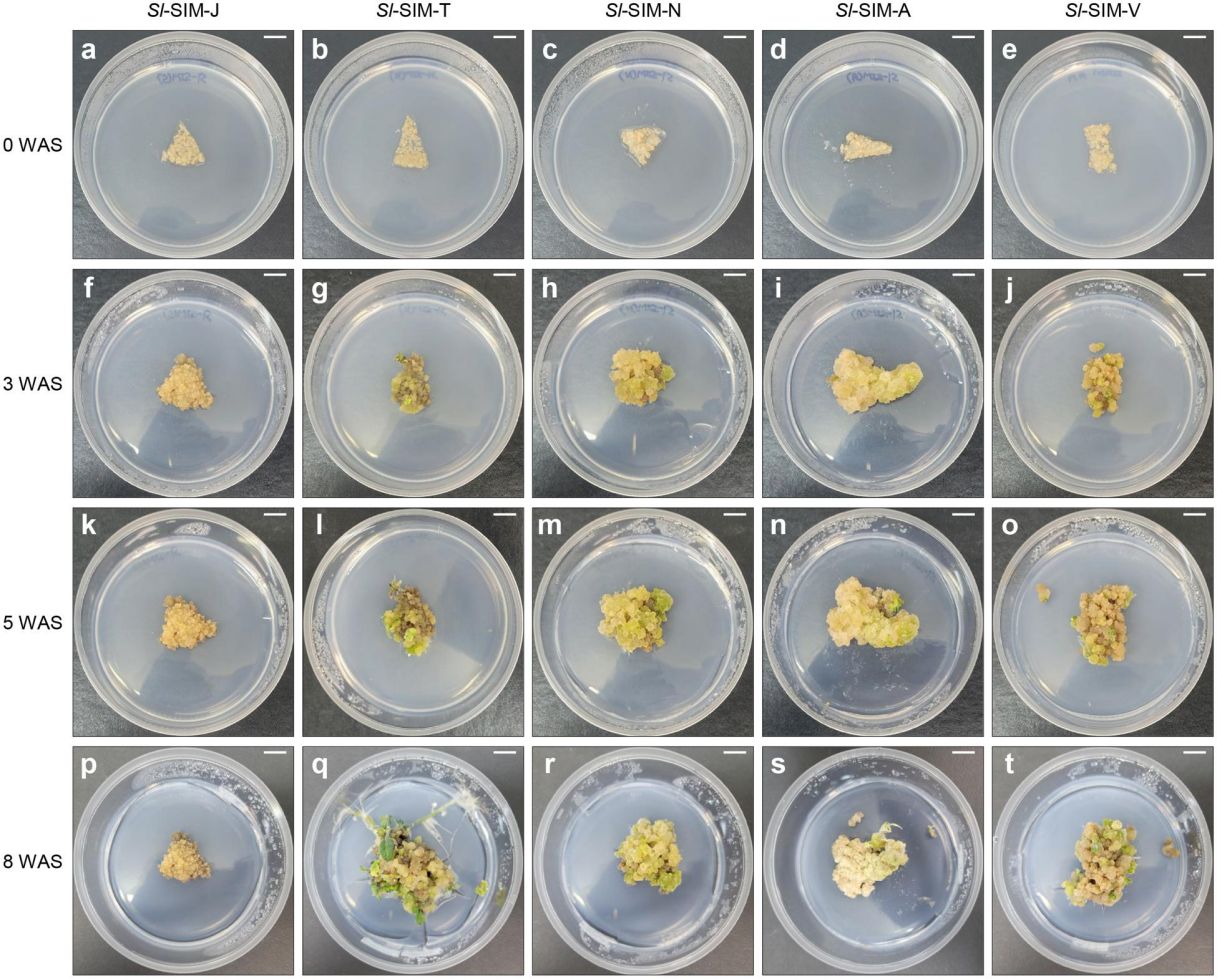
Shoot regeneration on five different shoot regeneration media. (**a**–**t**) Images showing *de novo* shoot organogenesis at the indicated number of WAS: 0 WAS (**a**–**e**), 3 WAS (**f**–**j**), 5 WAS (**k**–**o**), and 8 WAS (**p**–**t**). Bars = 1 cm

To induce shoot regeneration from microcalli grown in *Sl*-CIM-hCk, we placed alginate hydrogel on a solid medium for shoot regeneration [42, 43]. We examined the shoot regeneration efficiency on five different media: *Sl-*SIM-J [3], *Sl-*SIM-T [32], *Sl-*SIM-N [44], *Sl-*SIM-A [17], and *Sl-*SIM-V [45] (Fig. 4a–e). Microcalli on *Sl*-SIM-T showed *de novo* shoot regeneration at around 3 weeks after incubation on shoot regeneration medium (3 WAS) (Fig. 4f–J). This was earlier than shoot regeneration on other shoot induction medium (SIM) (Table 3). *Sl*-SIM-A and *Sl*-SIM-V showed a moderate shoot regeneration capacity, with shoot regeneration taking ∼5 weeks (Fig. 4k–o). In contrast, shoot regeneration efficiency was markedly low on *Sl*-SIM-J or *Sl*-SIM-N (Fig. 4p–t).

**Table 3.** Mean number of regenerated shoots from callus derived from *S. lycopersicum* cv. Micro-Tom protoplasts.

Medium	*Sl*-SIM-J	*Sl*-SIM-T	*Sl*-SIM-N	*Sl*-SIM-A	*Sl*-SIM-V
Number of regenerated shoots/hydrogel	1.33 ± 0.67	17.33 ± 2.60	ND	4.00 ± 1.52	11.33 ± 1.45

Protoplast hydrogels incubated in *Sl*-CIM-hCk were transferred to five different media for shoot regeneration. After 8 weeks of incubation, the number of regenerated shoots from each hydrogel was measured. Data represent the mean ± SEM of biological triplicates.

ND, not determined.

#### Protocol for de novo shoot regeneration from callus

1. Prepare 100-mm culture dishes containing *Sl*-SIM-T supplemented with 0.8% (w/v) agar.
2. Transfer alginate hydrogel onto the *Sl*-SIM-T using a sterilized spatula.
3. Seal the *Sl*-SIM-T plate with masking tape and incubate the plates at 25°C under LD conditions (70 µmol photons/m^2^/s) for 8 weeks.

### Plantlet formation and reproduction

**Figure 5:**
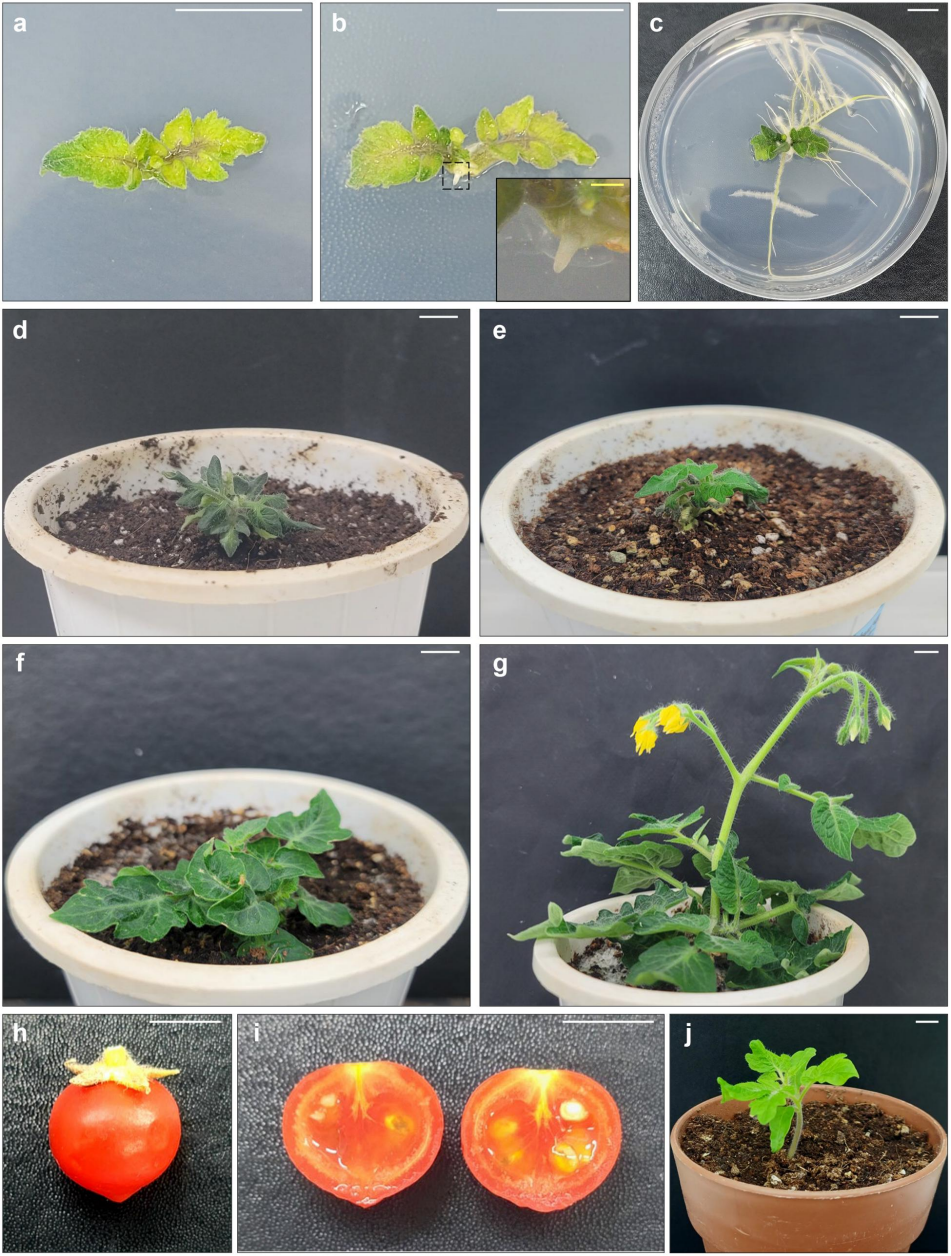
*De novo* root regeneration from regenerated shoots on *Sl*–RM. (**a**–**c**) Images showing *de novo* root organogenesis at the indicated number of weeks after incubation on *Sl*–RM (WAR): 0 WAR (**a**), 0.6 WAR (**b**), and 2 WAR (**c**). The boxed area is magnified in the inset image. (**d**–**g**) Images of regenerated plantlets at the indicated number of weeks after soil transfer (WAST): 0 WAST (**d**), 2 WAST (**e**), 4 WAST (**f**), and 8 WAST (**g**). (**h** and **i**) Images showing a whole (**h**) and longitudinal section (**i**) of a fruit produced by a protoplast-derived plantlet (R_0_) at 21 WAST. (**j**) Image of progeny (R_1_) at 3 weeks after sowing in soil. White bars = 1 cm; yellow bar = 1 mm

Regenerated shoots were excised from calli and subjected to *de novo* root organogenesis. We induced *de novo* root regeneration by incubating regenerated shoot explants on two different media: *Sl*-MS and *Sl*-RM (Table 4) [3]. Root regeneration efficiency measurements revealed that *Sl-*RM was more effective at promoting adventitious root formation, which occurred within 2 weeks of incubation (Fig. 5a–c).

**Table 4.** Rooting efficiency of regenerated shoots.

Medium	*Sl*-MS	*Sl*-RM
Percentage (%) of root-regenerated shoot explants	ND	61.11 ± 20.03

Regenerated shoots were excised from calli and transferred to one of two media for root regeneration. After 2 weeks of incubation, the percentage of root-regenerated explants relative to the total number of explants was calculated. Data represent the mean ± SEM of biological triplicates.

ND, not determined.

Next, we transplanted the regenerated plantlets into soil to continue their growth and reproductive development (Fig. 5d–g). The regenerated plantlets (R_0_) produced normal fruit progeny (R_1_) after soil transfer (Fig. 5h and i). R_1_ seeds were sown in soil, and the growth and development of R_1_ plants were monitored. Post-embryonic growth of R_1_ plants showed normal development (Fig. 5j), demonstrating that this protoplast regeneration protocol produces fertile plants and morphologically normal progeny.

#### De novo root organogenesis protocol

1. Prepare 100-mm culture dishes containing *Sl*-RM supplemented with 0.8% (w/v) agar.
2. Excise the basal parts of regenerated shoots and place the explants on *Sl*-RM.
*Note:* Callus tissue should be completely removed from explants as it inhibits *de novo* root regeneration.
3. Seal *Sl*-RM plates with masking tape and incubate plates at 25°C under LD conditions with 70 µmol photons/m^2^/s light intensity (cool-white fluorescent lamps) for 2 weeks.
4. Transplant regenerated plantlets (R_0_) to soil-filled pots.
5. Cover the pots with plastic wrap for 1 week.
6. Acclimate plantlets before removing the plastic wrap completely.
*Note:* Because regenerated plantlets are extremely sensitive to rapid environmental changes, progressive exposure to the growth environment is necessary.
7. Harvest R_1_ seeds.

## Discussion

*S. lycopersicum* cv. Micro-Tom is an important research model for *Solanum* species and has been widely used for basic research as well as biotechnological applications [19, 20]. Although several protoplast regeneration protocols have been developed for *Solanum* species, there has been no reliable Micro-Tom protoplast regeneration method. Here, we optimized a Micro-Tom protoplast regeneration method, which consists of five main steps: intact protoplast isolation, protoplast cell cycle reentry, microcallus formation, *de novo* shoot regeneration, and *de novo* root organogenesis. Each step was optimized by examining multiple culture conditions. In particular, we tried to determine the optimal culture medium since this can influence different species and culture methods differently.

Notably, *de novo* shoot organogenesis from microcalli is the major bottleneck in Micro-Tom protoplast regeneration. In general, previous studies suggested that high cytokinin concentrations are important for promoting shoot regeneration [29], whereas high concentrations of auxin frequently induce callus browning with lower shoot regeneration rates [32]. Consistent with this, our results indicate that the cytokinin concentration in the shoot regeneration medium is somewhat correlated with shoot regeneration rates. In particular, *Sl*-SIM-T, which contains relatively high cytokinin concentration, showed the best performance in shoot regeneration, compared to the others examined. However, cytokinin is not the only factor that determines shoot regeneration rates in Micro-Tom protoplast-derived calli. For instance, *Sl*-SIM-N containing the same *trans*-zeatin concentration as *Sl*-SIM-T did not efficiently induce shoot regeneration in our conditions. This observation suggests that the optimal cytokine/auxin ratio, higher sucrose concentration, and/or lower gibberellin level in *Sl*-SIM-T may also contribute to enhancing shoot regeneration in Micro-Tom protoplast-derived calli. Considering that *Sl*-SIM-N was successfully used for shoot regeneration of callus derived from protoplasts of the tomato cultivar Red Setter [17], Micro-Tom probably requires a distinct culture medium possibly due to its altered hormonal balances, such as lower brassinosteroid and gibberellin levels. Thus, our protocol could be a good option for various tomato varieties, but its applicability needs to be evaluated, because cell and tissue culture methods usually vary, depending on plant variety and species.

This study suggests several differences compared to previous tomato cell culture methods. A series of cytokinin-rich culture media, *Sl*-PIM-hCk, *Sl*-CIM-hCk, and *Sl*-SIM-T, are the optimal media for Micro-Tom protoplast regeneration. In addition, transferring an alginate gel, instead of individual microcalli, onto SIM also enhances shoot regeneration efficiency, possibly because physical damage to calli is minimized [42, 43]. The process following *de novo* shoot regeneration was also straightforward. Regenerated shoot explants exhibited high root regeneration efficiency on the *Sl*-RM medium. Protoplast-derived regenerated plantlets underwent normal growth and development, producing morphologically normal progeny. However, considering that the time period required for shoot regeneration is still long, exogenous chemical treatment to enhance shoot regeneration might be advantageous.

This protocol allows Micro-Tom protoplast regeneration with high efficiency and reproducibility within 5 months and is expected to work for several other tomato varieties [33, 46]. We believe that this study will accelerate fundamental research covering cell division, pluripotency acquisition, and tissue regeneration as well as a variety of biotechnological applications, including cell and genome engineering and nanomaterial applications, in various *Solanum* species and tomato cultivars.

## Conclusions

We determined source tissues for protoplast isolation and culture conditions for each step of protoplast regeneration. The protocol comprises five main steps that can be completed within 5 months, allowing fundamental studies and biotechnological applications for crop improvement.

## Supplementary Material

bpae008_Supplementary_Data

## Data Availability

All data generated or analyzed during this study are included in this published article and its online supplementary materials.

## References

[1] Ikeuchi M , Favero DS, Sakamoto Y et al Molecular mechanisms of plant regeneration. Annu Rev Plant Biol 2019;70:377–406. 30786238 10.1146/annurev-arplant-050718-100434

[2] Roest S , Gilissen LJW. Plant-regeneration from protoplasts—a literature-review. Acta Bot Neerl 1989;38:1–23.

[3] Jeong YY , Lee H-Y, Kim SW et al Optimization of protoplast regeneration in the model plant *Arabidopsis thaliana*. Plant Methods 2021;17:21.16. 33622383 10.1186/s13007-021-00720-x PMC7901198

[4] Kiełkowska A , Adamus A. An alginate-layer technique for culture of *Brassica oleracea* L. protoplasts. In Vitro Cell Dev Biol Plant 2012;48:265–73. 22593638 10.1007/s11627-012-9431-6 PMC3337407

[5] Grzebelus E , Szklarczyk M, Baranski R. An improved protocol for plant regeneration from leaf- and hypocotyl-derived protoplasts of carrot. Plant Cell Tiss Organ Cult 2012;109:101–9.

[6] Barceló M , Wallin A, Medina JJ et al Isolation and culture of strawberry protoplasts and field evaluation of regenerated plants. Sci Hortic-Amsterdam 2019;256:108552.

[7] Damm B , Willmitzer L. Regeneration of fertile plants from protoplasts of different *Arabidopsis thaliana* genotypes. Molec Gen Genet 1988;213:15–20.

[8] Zhang YX , Iaffaldano B, Qi YP. CRISPR ribonucleoprotein-mediated genetic engineering in plants. Plant Commun 2021;2:100168. 33898980 10.1016/j.xplc.2021.100168 PMC8060726

[9] Lin CS , Hsu CT, Yang LH et al Application of protoplast technology to CRISPR/Cas9 mutagenesis: from single-cell mutation detection to mutant plant regeneration. Plant Biotechnol J 2018;16:1295–310. 29230929 10.1111/pbi.12870 PMC5999315

[10] Andreasson E , Kieu NP, Zahid MA et al Invited Mini-Review Research Topic: utilization of Protoplasts to Facilitate Gene Editing in Plants: schemes for Shoot Regeneration From Tissues and Protoplasts of Potato and Rapeseed: implications of Bioengineering Such as Gene Editing of Broad-Leaved Plants. Front Genome Ed 2022;4:780004. 35845346 10.3389/fgeed.2022.780004 PMC9276966

[11] Woo JW , Kim J, Kwon SI et al DNA-free genome editing in plants with preassembled CRISPR-Cas9 ribonucleoproteins. Nat Biotechnol 2015;33:1162–4. 26479191 10.1038/nbt.3389

[12] Lin CS , Hsu CT, Yuan YH et al DNA-free CRISPR-Cas9 gene editing of wild tetraploid tomato using protoplast regeneration. Plant Physiol 2022;188:1917–30. 35088855 10.1093/plphys/kiac022 PMC8968427

[13] González MN , Massa GA, Andersson M et al Reduced enzymatic browning in potato tubers by specific editing of a polyphenol oxidase gene ribonucleoprotein complexes delivery of the CRISPR/Cas9 System. Front Plant Sci 2019;10:1649. 31998338 10.3389/fpls.2019.01649 PMC6962139

[14] Lee MH , Lee J, Choi SA et al Efficient genome editing using CRISPR-Cas9 RNP delivery into cabbage protoplasts via electro-transfection. Plant Biotechnol Rep 2020;14:695–702.

[15] Najafi S , Bertini E, D'Inca E et al DNA-free genome editing in grapevine using CRISPR/Cas9 ribonucleoprotein complexes followed by protoplast regeneration. Hortic Res-England 2023;10:uhac240. 10.1093/hr/uhac240 PMC10108004 37077374

[16] Yu J , Tu L, Subburaj S et al Simultaneous targeting of duplicated genes in protoplasts for flower color modification via CRISPR-Cas9 ribonucleoproteins. Plant Cell Rep 2021;40:1037–45. 32959126 10.1007/s00299-020-02593-1

[17] Liu Y , Andersson M, Granell A et al Establishment of a DNA-free genome editing and protoplast regeneration method in cultivated tomato (*Solanum lycopersicum*). Plant Cell Rep 2022;41:1843–52. 35773498 10.1007/s00299-022-02893-8 PMC9395478

[18] Campos MD , Felix MDR, Patanita M et al High throughput sequencing unravels tomato-pathogen interactions towards a sustainable plant breeding. Hortic Res 2021;8:171. 34333540 10.1038/s41438-021-00607-x PMC8325677

[19] Marti E , Gisbert C, Bishop GJ et al Genetic and physiological characterization of tomato cv. Micro-Tom. J Exp Bot 2006;57:2037–47. 16687436 10.1093/jxb/erj154

[20] Meissner R , Jacobson Y, Melamed S et al A new model system for tomato genetics. Plant J 1997;12:1465–72.

[21] Brooks C , Nekrasov V, Lippman ZB et al Efficient gene editing in tomato in the first generation using the clustered regularly interspaced short palindromic repeats/CRISPR-associated9 system. Plant Physiol 2014;166:1292–7. 25225186 10.1104/pp.114.247577 PMC4226363

[22] Xu C , Liberatore KL, MacAlister CA et al A cascade of arabinosyltransferases controls shoot meristem size in tomato. Nat Genet 2015;47:784–92. 26005869 10.1038/ng.3309

[23] Xu C , Park SJ, Van Eck J et al Control of inflorescence architecture in tomato by BTB/POZ transcriptional regulators. Genes Dev 2016;30:2048–61. 27798848 10.1101/gad.288415.116 PMC5066612

[24] Nonaka S , Arai C, Takayama M et al Efficient increase of ɣ-aminobutyric acid (GABA) content in tomato fruits by targeted mutagenesis. Sci Rep 2017;7:7057. 28765632 10.1038/s41598-017-06400-y PMC5539196

[25] Li R , Li R, Li X et al Multiplexed CRISPR/Cas9-mediated metabolic engineering of γ-aminobutyric acid levels in *Solanum lycopersicum*. Plant Biotechnol J 2018;16:415–27. 28640983 10.1111/pbi.12781 PMC5787826

[26] Ortigosa A , Gimenez-Ibanez S, Leonhardt N et al Design of a bacterial speck resistant tomato by CRISPR/Cas9-mediated editing of *SlJAZ2*. Plant Biotechnol J 2019;17:665–73. 30183125 10.1111/pbi.13006 PMC6381780

[27] Tran MT , Doan DTH, Kim J et al CRISPR/Cas9-based precise excision of *SlHyPRP1* domain(s) to obtain salt stress-tolerant tomato. Plant Cell Rep 2021;40:999–1011. 33074435 10.1007/s00299-020-02622-z

[28] Shimatani Z , Kashojiya S, Takayama M et al Targeted base editing in rice and tomato using a CRISPR-Cas9 cytidine deaminase fusion. Nat Biotechnol 2017;35:441–3. 28346401 10.1038/nbt.3833

[29] Reed KM , Bargmann BOR. Protoplast Regeneration and Its Use in New Plant Breeding Technologies. Front Genome Ed 2021;3:734951. 34713266 10.3389/fgeed.2021.734951 PMC8525371

[30] Niedz RP , Rutter SM, Handley LW et al Plant regeneration from leaf protoplasts of six tomato cultivars. Plant Sci 1985;39:199–204.

[31] Hossain M , Imanishi S, Egashira H. An improvement of tomato protoplast culture for rapid plant-regeneration. Plant Cell Tiss Organ Cult 1995;42:141–6.

[32] Tan ML , Rietveld EM, van Marrewijk GA et al Regeneration of leaf mesophyll protoplasts of tomato cultivars (*L. esculentum*): factors important for efficient protoplast culture and plant regeneration. Plant Cell Rep 1987;6:172–5. 24248643 10.1007/BF00268470

[33] Mühlbach HP. Different regeneration potential of mesophyll protoplasts from cultivated and a wild species of tomato. Planta 1980;148:89–96. 24311271 10.1007/BF00385447

[34] Gleddie S , Keller WA, Poysa V. Plant regeneration from stem cortex protoplasts of a tomato hybrid. Plant Cell Rep 1989;8:21–4. 24232588 10.1007/BF00735770

[35] Fossi M , Amundson K, Kuppu S et al Regeneration of *Solanum tuberosum* plants from protoplasts induces widespread genome instability. Plant Physiol 2019;180:78–86. 30792232 10.1104/pp.18.00906 PMC6501065

[36] Binding H , Nehls R. Regeneration of isolated protoplasts to plants in *Solanum-Dulcamara* L. Z Pflanzenphysiol 1977;85:279–80.

[37] Grzebelus E , Skop L. Effect of β-lactam antibiotics on plant regeneration in carrot protoplast cultures. In Vitro Cell Dev Biol Plant 2014;50:568–75. 25298730 10.1007/s11627-014-9626-0 PMC4182649

[38] Moon KB , Park JS, Park SJ et al A more accessible, time-saving, and efficient method for in vitro plant regeneration from potato protoplasts. Plants 2021;10:781. 33923378 10.3390/plants10040781 PMC8071491

[39] Van den Broeck L , Schwartz MF, Krishnamoorthy S et al Establishing a reproducible approach to study cellular functions of plant cells with 3D bioprinting. Sci Adv 2022;8:eabp9906. 36240264 10.1126/sciadv.abp9906 PMC9565790

[40] Potrykus I , Shillito RD, Protoplasts: isolation, culture, plant-regeneration. Method Enzymol 1986;118:549–78.

[41] Tomiczak K , Mikuła A, Sliwinska E et al Autotetraploid plant regeneration by indirect somatic embryogenesis from leaf mesophyll protoplasts of diploid *Gentiana decumbens* L.f. In Vitro Cell Dev Biol Plant 2015;51:350–9. 26097374 10.1007/s11627-015-9674-0 PMC4471314

[42] Dovzhenko A , Dal Bosco C, Meurer J et al Efficient regeneration from cotyledon protoplasts in *Arabidopsis thaliana*. Protoplasma 2003;222:107–11. 14513316 10.1007/s00709-003-0011-9

[43] Pati PK , Sharma M, Ahuja PS. Extra thin alginate film: an efficient technique for protoplast culture. Protoplasma 2005;226:217–21. 16244810 10.1007/s00709-005-0096-4

[44] Nicolia A , Proux-Wera E, Ahman I et al Targeted gene mutation in tetraploid potato through transient TALEN expression in protoplasts. J Biotechnol 2015;204:17–24. 25848989 10.1016/j.jbiotec.2015.03.021

[45] Valvekens D , Van Montagu M, Van Lijsebettens M. *Agrobacterium tumefaciens*-mediated transformation of *Arabidopsis thaliana* root explants by using kanamycin selection. Proc Natl Acad Sci U S A 1988;85:5536–40. 16593964 10.1073/pnas.85.15.5536 PMC281793

[46] Morgan A , Cocking EC. Plant regeneration from protoplasts of *Lycopersicon esculentum* Mill. Z Pflanzenphysiol 1982;106:97–104.

