# Supplementary material for: Efficient regeneration of protoplasts from Solanum lycopersicum cultivar Micro-Tom
Source: Biol Methods Protoc. 2024 Feb 6;9(1):bpae008. doi: 10.1093/biomethods/bpae008 (PMC10898868; doi:10.1093/biomethods/bpae008)
Supplement: bpae008_Supplementary_Data [file bpae008_supplementary_data.pdf]

Supplementary Tables

Reagents used in this study

- Viscozyme L (Novozymes, SP079)
- Pectinex Ultra SP-L (Novozymes, 1043-70)
- Celluclast 1.5L (Novozymes, SP011)
- MES monohydrate (Duchefa Biochemie, M1503)
- $CaCl_2$ (Junsei, 18235-0301)
- D-mannitol (Junsei, 70220-0301)
- NaOH (Sigma-Aldrich, S8045)
- Sucrose (Sigma-Aldrich, S1888)
- D-glucose (Sigma-Aldrich, G7021)
- Sodium alginate (Sigma-Aldrich, A0682)
- Plant agar (Duchefa Biochemie, P1001)
- Gamborg B5 medium including vitamins (Duchefa Biochemie, G0210)
- Gamborg B5 medium (Duchefa Biochemie, G0209)
- Kao and Michayluk Vitamin Solution (Sigma-Aldrich, K3129)
- Folic acid (Duchefa Biochemie, F0608)
- Casein hydrolysate (Duchefa Biochemie, C1301)
- Murashige & Skoog medium including vitamins (Duchefa Biochemie, M0222)
- Myo-inositol (Duchefa Biochemie, I0609)
- 6-Benzylaminopurine (6-BAP) (Duchefa Biochemie, B0904)
- α -Naphtalene acetic acid (α -NAA) (Duchefa Biochemie, N0903)
- Indole-3-butyric acid (IBA) (Duchefa Biochemie, I0902)
- Indole-3-acetic acid (IAA) (Duchefa Biochemie, I0901)
- 6-(γ,γ-Dimethylallylamino)purine (2-ip) (Sigma-Aldrich, D7674)
- 2,4-Dichlorophenoxyacetic acid (2,4-D) (Duchefa Biochemie, D0911)
- *trans*-Zeatin (GoldBio, Z-105)
- Gibberellic acid 3 (GA3) (Duchefa Biochemie, G0907)
- Ethanol (Merck, 8.18760)
- Triton X-100 (Sigma-Aldrich, T9284)

Supplementary Table 1. Chemicals used in this study

Equipment used in this study

- Pipette-aid (Drummond, HDR-4-000-201)
- 10 mL serological pipette (SPL, 91010)
- 5 mL syringe (Kovax)
- 50 mL syringe (Kovax)
- 0.2 μm syringe sterilization filter (Sartorius, 16534K)
- 40 μm cell strainer (SPL, 93040)
- 100 mm culture dish (SPL, 310100)
- 90 mm plate (SPL, 20100)
- 60 mm plate (SPL, 20060)
- 3M micropore tape (3M, 1530-0)
- Parafilm (Sigma-Aldrich, PM996)
- 14 mL round-bottomed tube (SPL, 40014)
- Hemocytometer (Marienfeld)
- Shaker (Vision Scientific, VS-201D)
- Optical microscope (Olympus, IX53)
- Stereoscopic microscope (Leica, Stereozoom S9i)
- Table-top centrifuge (Hanil, Combi 514R)
- Autoclave (Vision Scientific, VS-1221)
- Versatile environmental test chamber (Panasonic, MLT-352H-PK)
- Double room incubator (Hanbaek Scientific Technology, HB-201MS-2R)
- Plant growth chamber (Vision Scientific, VS-91G09M-600)
- Clean bench (Vision Scientific, VS-1400LHN)
- Stainless-steel spatula (Scilab, SL.sap7012)
- Stainless-steel forceps (Scilab, SL.For7076)
- Stainless-steel Scalpel blade (Feather, HFE-SB10)
- Stainless-steel Scalpel handle (Feather, 72040-03)

Supplementary Table 2. Equipment used in this study

Supplementary Figures

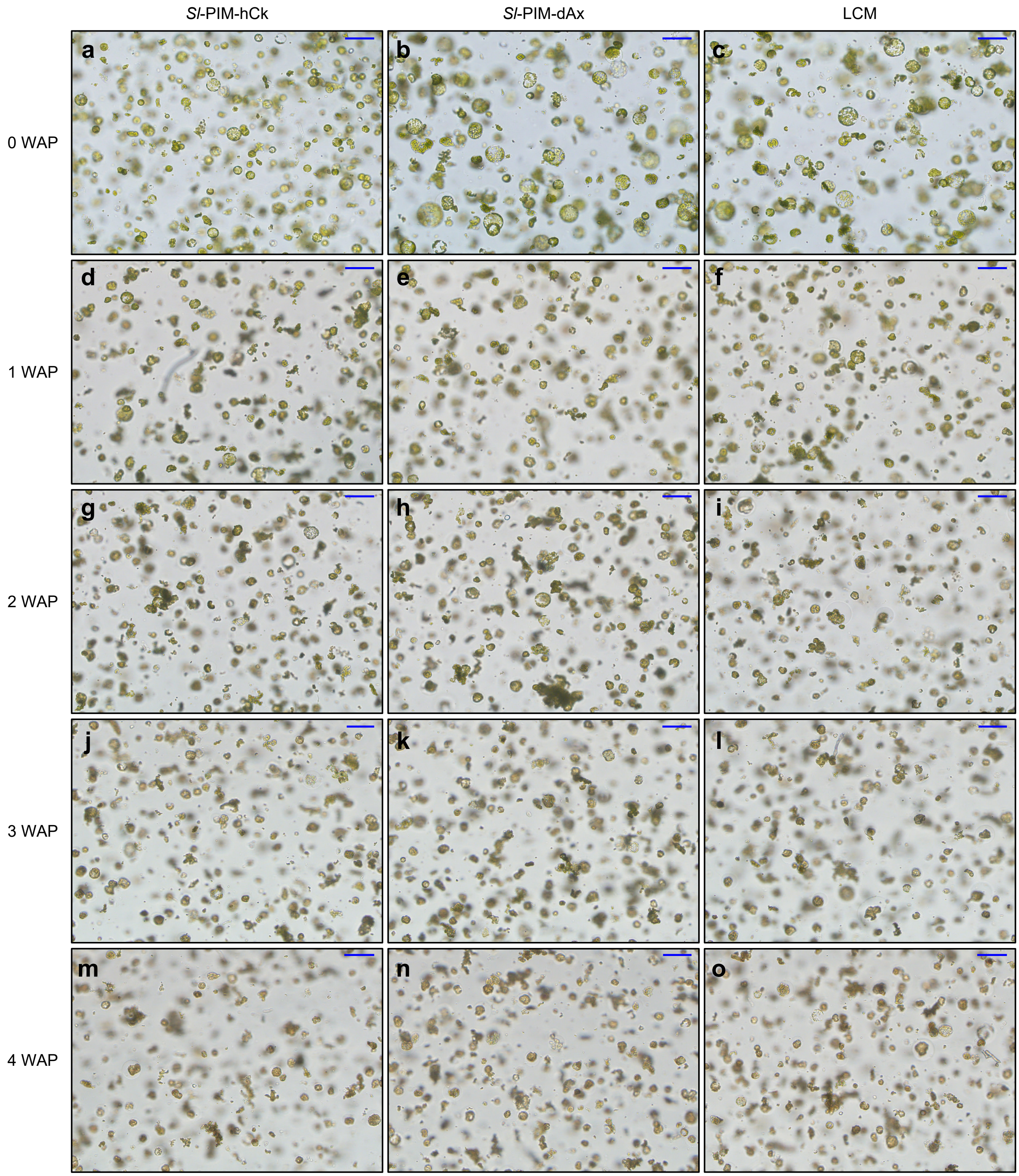

Supplementary Figure 1. Division of cotyledon-derived protoplasts in three different protoplast proliferation media. (**a**–**o**) Protoplast images were taken at the indicated time points (weeks) after incubation in protoplast proliferation medium (WAP): 0 WAP (**a**–**c**), 1 WAP (**d**–**f**), 2 WAP (**g**–**i**), 3 WAP (**j**–**l**), and 4 WAP (**m**–**o**). Scale bars = 100 μm.

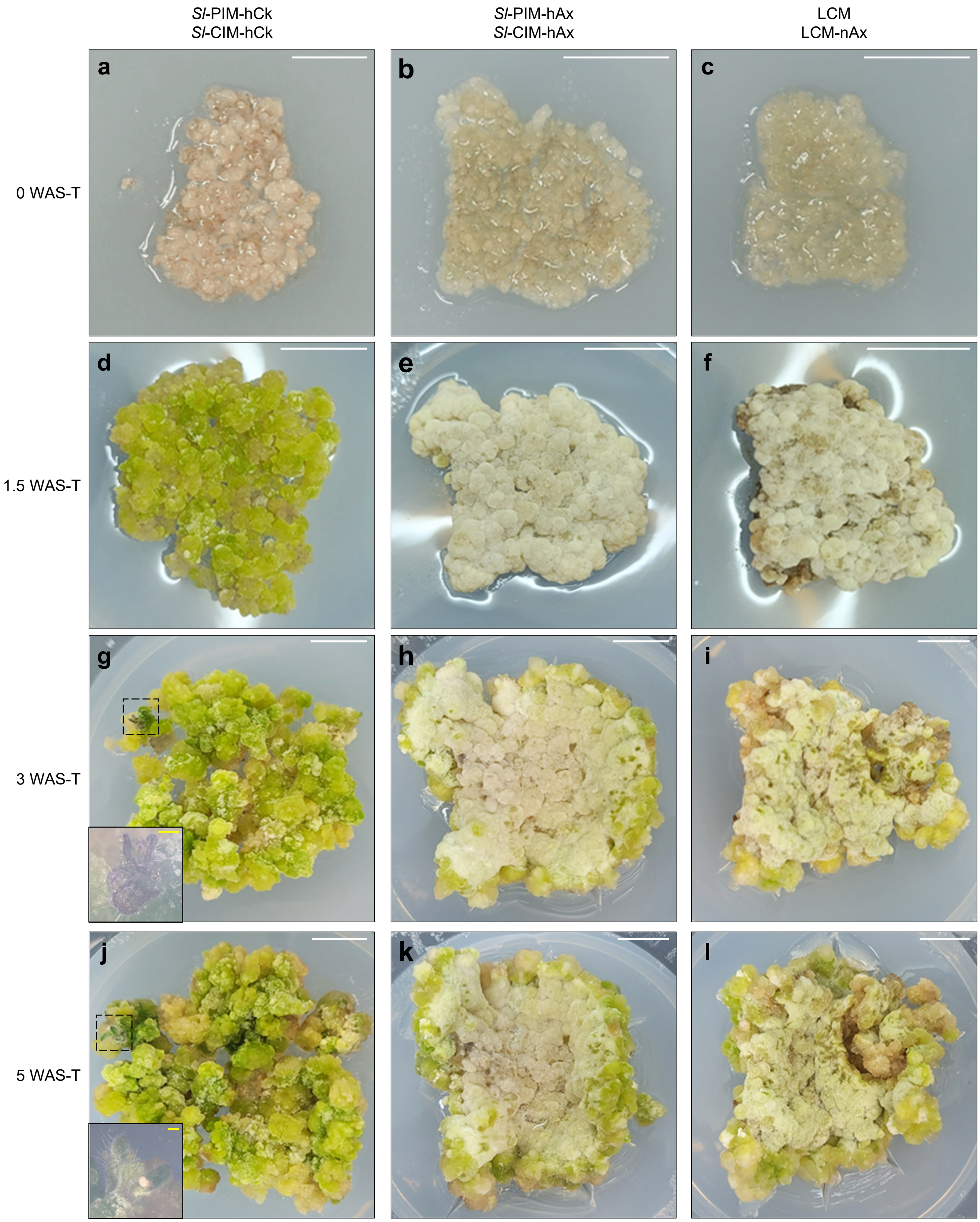

Supplementary Figure 2. Shoot regeneration from microcallus produced in three different pairs of protoplast and callus proliferation media. (**a**–**l**) Images for *de novo* shoot organogenesis were taken at the indicated time points (weeks) after incubation on shoot induction medium-T (WAS-T): 0 WAS-T (**a**–**c**), 1.5 WAS-T (**d**–**f**), 3 WAS-T (**g**–**i**), 5 WAS-T (**j**–**l**). In (**g**) and (**j**), the dashed box region is magnified in the inset. White bars = 1 cm; Yellow bars = 1 mm.
